# Editorial: Study on immune mechanism and immune intervention in connective tissue diseases

**DOI:** 10.3389/fmed.2025.1610785

**Published:** 2025-07-03

**Authors:** Giuseppe Murdaca, Wenru Su, Zhiming Lin, Christina Adamichou, Patrizia Leone, Mariusz Sikora, Francesca Paladin

**Affiliations:** ^1^Department of Internal Medicine, University of Genova, Viale Benedetto XV, Genoa, Italy; ^2^Allergology and Clinical Immunology Unit, San Bartolomeo Hospital, Sarzana, Italy; ^3^State Key Laboratory of Ophthalmology, Zhongshan Ophthalmic Center, Sun Yat-Sen University, Guangdong Provincial Key Laboratory of Ophthalmology and Visual Science, Guangzhou, China; ^4^Division of Rheumatology, Third Affiliated Hospital of Sun Yat-Sen University, Guangzhou, China; ^5^Fourth Department of Internal Medicine, Hippokration Hospital, Aristotle University of Thessaloniki, Thessaloniki, Greece; ^6^Department of Interdisciplinary Medicine, School of Medicine, ‘Aldo Moro' University of Bari, Bari, Italy; ^7^National Institute of Geriatrics, Rheumatology and Rehabilitation, Warsaw, Poland; ^8^Elderly and Disabeld Department, San Paolo Hospital, Savona, Italy

**Keywords:** connective tissue diseases, immune responses, immunotherapy, cytokines, immunosuppressive agents

Connective tissue diseases (CTDs) are a group of autoimmune disorders that primarily affect the connective tissues, including the skin, joints, blood vessels, and internal organs. This group of diseases include conditions such as rheumatoid arthritis (RA), systemic lupus erythematosus (SLE), systemic sclerosis (SS), and mixed connective tissue disease (MCTD). They occur when the body's immune system mistakenly targets and attacks its own tissues. The immune mechanisms involved in CTDs are complex and vary depending on the specific disease, but generally, several common immune processes are implicated.

Among the shared immunological mechanisms in CTDs, the production of autoantibodies specific to each disease plays a central role in diagnosis. For example, rheumatoid factor (RF) and anti-citrullinated protein antibodies (ACPA) are strongly related to RA, while antinuclear antibodies (ANA) and anti-double-stranded DNA (dsDNA) are associated with SLE. Anti-topoisomerase I and anti-centromere antibodies are markers for SS, and Anti-U1 RNP antibodies are found in MCTD (1). Recently, anti-Ro60 and anti-Ro52 antibodies have been identified as clinically relevant to the severity of diseases such as SS and SLE (2). In the case of anti-U1RNP+ MCTDs, autoantibodies against the motor neuron complex (anti-SMN antibodies) may help define not only the clinical severity in terms of multi-organ involvement but also the disease's phenotypic characteristics, thus offering a useful diagnostic and prognostic tool (3).

T-cell involvement, particularly CD4+ T-helper cells, is also crucial in the pathogenesis of CTDs. These cells initiate and sustain autoimmune responses by producing and releasing specific inflammatory mediators such as interleukins (IL-1, IL-6) and tumor necrosis factor (TNF), which recruit additional immune cells (macrophages, neutrophils) to the site of tissue damage. Yixian et al. demosntrated that elevated CD28 level on CD4-secreting CD39+ regulatory T cells increases the risk of SS, while elevated CD3 level on CD39+ CD8+ T cells decreases it. Furthermore, increased expression of CD24 on memory B cells and CD27 on IgD+ CD24+ B cells promotes the development of SS, while increased CD38 on IgD+ CD38+ B cells reduces it. Notably, efferocytosis is the process by which apoptotic cells are removed by phagocytic cells. It can be thought of as the “burial of dead cells.” Defective efferocytosis has been demonstrated in several inflammatory diseases including RA and SLE. The resulting inflammation and cellular necrosis releases the cell contents sustaining the chronic inflammation. Lofaro et al. developed a systematic review that includes 1,003 papers confirming the ever-increasing scientific attention on this Research Topic supported by the constant increase in the number of publications.

Mo et al. emphasized the regulatory role of competing endogenous RNAs (ceRNAs) in the pathogenesis and treatment of SLE. However, many CTDs have a genetic component, with specific genetic variants (e.g., HLA genes) linked to an increased risk of developing these diseases. In particular, Ivanova et al. confirmed that HLA-B^*^08:01 allele was the primary risk factor for early-onset myastenia gravis (MG) and HLA-DRB1^*^15:01 allele for late-onset MG. Furthermore, the expression of HLA-A^*^25, HLA-B^*^40:01 and HLA-DRB1^*^16 predisposes to a higher risk of developing thymoma-associated MG.

Vitamin D receptor (VDR) polymorphisms have also been associated with susceptibility to diseases such as SLE, primary Sjögren's syndrome (pSS), and RA (4).

Immunotherapy for CTDs aims to modulate the immune system to reduce inflammation, prevent tissue damage, and improve symptoms. The goal is to suppress the overactive immune response or target specific molecules involved in the inflammatory process.

The first line of treatment often involves corticosteroids, which are powerful anti-inflammatory drugs that suppress the immune system. While effective, corticosteroids have significant side effects, especially with long-term use, such as weight gain, osteoporosis, diabetes, and an increased risk of infection. In addition to corticosteroids, immunosuppressive drugs are commonly used. These include methotrexate (for RA), azathioprine (for SLE and RA), cyclophosphamide, and mycophenolate mofetil (for lupus nephritis).

TNF-α inhibitors, such as infliximab, adalimumab, and etanercept, are biologic therapies used to treat various autoimmune diseases, including several CTDs. By inhibiting TNF, these drugs reduce inflammation and prevent further tissue and organ damage.

Janus Kinase inhibitors (JAK inhibitors) are another class of medications that target specific enzymes involved in immune response and inflammation. These inhibitors, which include tofacitinib, baricitinib, upadacitinib, and filgotinib, have become an important treatment option for patients with diseases difficult to manage with traditional therapies. They help control inflammation, reduce disease activity, and prevent further organ damage.

B-cell depletion therapy is an advanced treatment used in several autoimmune and connective tissue diseases. The most widely used B-cell depletion therapy involves monoclonal antibodies that target CD20, a protein on the surface of most B cells. Rituximab and ofatumumab are the main monoclonal antibodies used for this purpose (5). These therapies reduce the number of active B cells in the body, thereby reducing autoimmune activity. In the context of advanced cellular therapies, mesenchymal stem cells (MSCs) and chimeric antigen receptor T cells (CAR-T cells) represent two innovative and promising approaches for the management of scleroderma (Chen et al.).

Monoclonal antibodies are also a key part of modern treatments for connective tissue diseases. They offer targeted therapies that provide better disease control with fewer side effects compared to traditional treatments. Examples include rituximab, abatacept, belimumab, tocilizumab, and secukinumab, which are used in conditions like lupus, rheumatoid arthritis, scleroderma, and vasculitis (6).

Gene therapy holds great promise for CTDs by targeting the underlying genetic causes of these diseases, correcting immune dysfunction, and promoting tissue repair. Although gene therapy for CTDs is still in its early stages, ongoing clinical trials and preclinical studies are investigating approaches such as CRISPR-Cas9 gene editing to modify immune cell behavior in diseases like lupus and rheumatoid arthritis (7).
